# Metastatic Adnexal Adenocarcinoma: A Case Report of Successful Treatment With Carboplatin and Paclitaxel

**DOI:** 10.7759/cureus.78067

**Published:** 2025-01-27

**Authors:** João N Soares, Victoria Guiote, Jose C Cardoso, Edgar Pratas, Joana Calvão

**Affiliations:** 1 Department of Dermatology, University Hospital of Coimbra, Coimbra, PRT; 2 Faculty of Medicine, University of Coimbra, Coimbra, PRT; 3 Department of Dermatology, Hospital Santo André, Leiria, PRT; 4 Department of Oncology, University Hospital of Coimbra, Coimbra, PRT

**Keywords:** adnexal adenocarcinoma, carboplatin, case report, chemotherapy, metastasis, paclitaxel

## Abstract

Metastatic adnexal adenocarcinoma not otherwise specified (AANOS) is a rare and aggressive tumor with little evidence about treatment options. We describe a case of metastatic AANOS that showed a positive response to carboplatin and paclitaxel. This response is novel in the current medical literature. We report the case of a 72-year-old male with no significant past medical history, presenting with a 4 cm occipito-temporal skin mass. A cutaneous biopsy and staging confirmed the diagnosis of primary cutaneous AANOS, with no evidence of metastasis. The patient underwent radical surgical excision followed by adjuvant radiotherapy. After 44 months of follow-up, the disease recurred locally and progressed with pulmonary, mediastinal, and bone metastases. The patient was started on a systemic chemotherapy regimen of carboplatin and paclitaxel. A near-complete response was observed after three months, which was sustained after one year of follow-up. Given the rarity of these tumors, no treatment guidelines are available, and evidence is based on single case reports and a few case series. A combination of carboplatin and paclitaxel provides a strong inhibition of cell mitosis and allows for the administration of lower doses at a higher frequency, thus balancing efficacy with reduced toxicity. Combination carboplatin and paclitaxel can be an effective treatment for metastatic AANOS.

## Introduction

Adnexal adenocarcinoma not otherwise specified (AANOS) is a rare and aggressive form of skin cancer that arises from the sweat glands [1,2]. It is known to recur and metastasize in approximately 12% [3] to 53% [4] of cases, with a poor prognosis, posing significant challenges for clinicians. The overall survival (OS) and recurrence-free survival (RFS) rates at five years are 62.4% and 47.4%, respectively [3]. However, given the low prevalence, there is little evidence about follow-up strategies and treatment options, and no guidelines are available [1,2]. We describe a case of metastatic AANOS that showed a significant response to carboplatin and paclitaxel. From what we were able to find, this therapeutic approach is novel in the current medical literature.

## Case presentation

**Figure 1:**
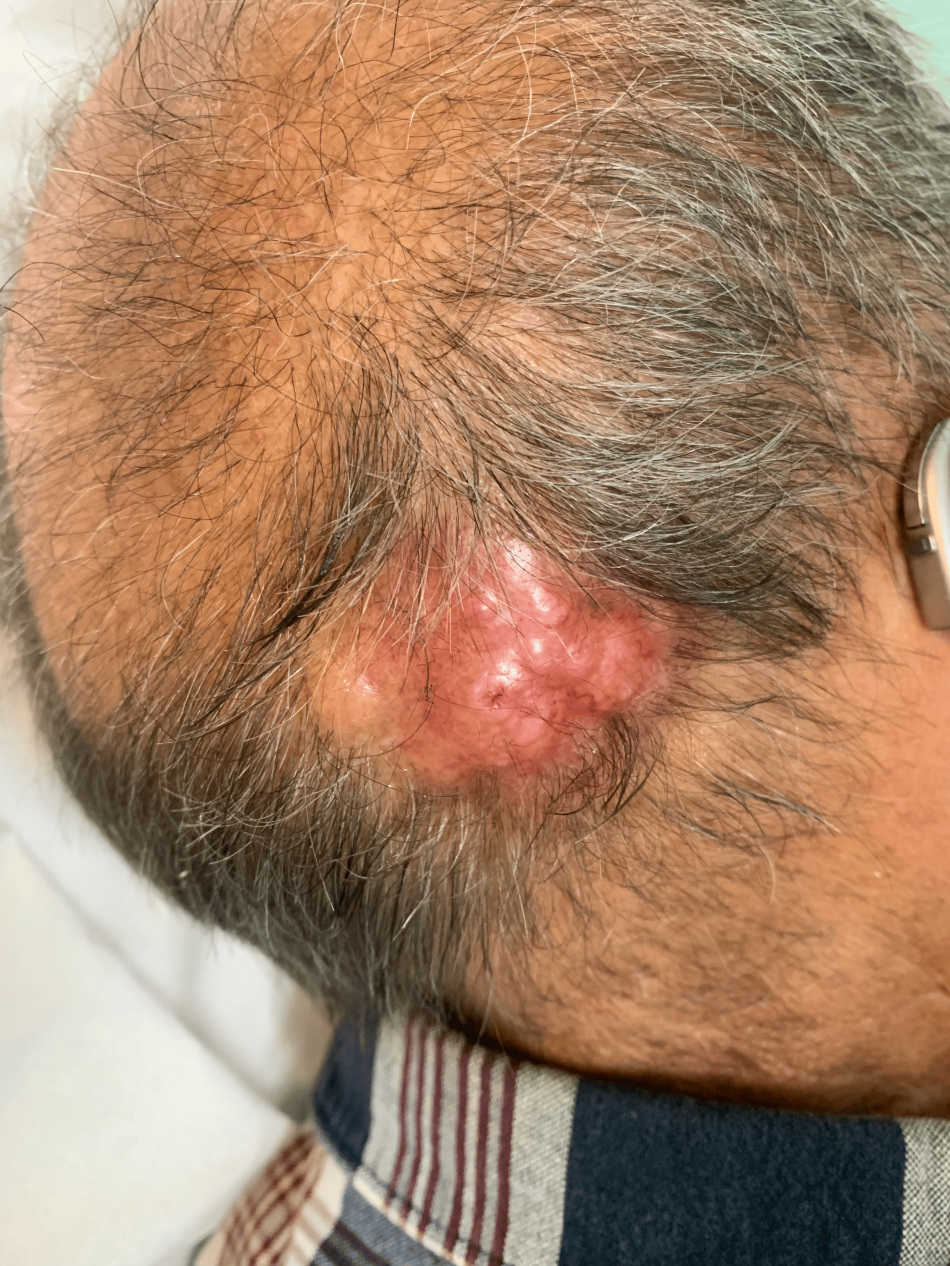
Clinical image of the primary tumor located in the right occipito-temporal region. The lesion was a 4 cm firm, irregularly shaped mass with an erythematous surface. On palpation, it was adherent to underlying structures but without evidence of ulceration or overt invasion into surrounding tissues.

A 72-year-old man with a good performance status (Eastern Cooperative Oncology Group Performance Status, ECOG-PS, 0) and no significant past medical history presented with a 4 cm right occipito-temporal mass (Figure 1). Although the mass was clinically adherent to the bone on palpation, there was no evidence of skull invasion on computed tomography (CT) imaging.

**Figure 2:**
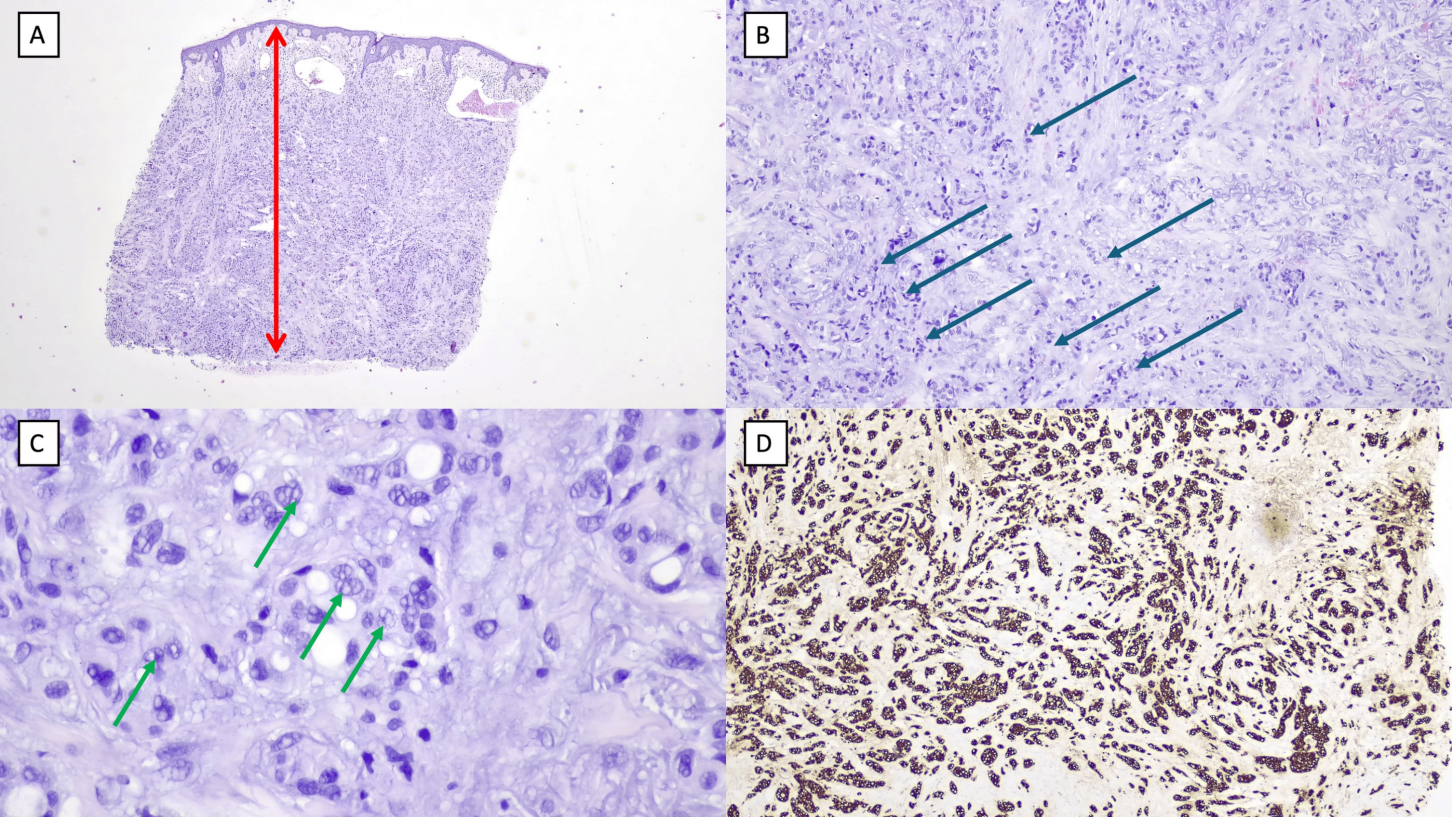
Histological sections of a primary right occipito-temporal nodule biopsy. (A) H&E, ×50: Low magnification view showing neoplasia with an infiltrative pattern throughout the full thickness of the dermis (red arrow). (B) H&E, ×100: Intermediate magnification illustrating cords of atypical cells with marked nuclear pleomorphism (blue arrows). (C) H&E, ×200: High magnification displaying cells with vacuoles, indicating areas of ductal differentiation (green arrows). (D) Immunohistochemistry: Intense and diffuse positivity for cytokeratin 7. CK20, TTF-1, and CDX2 were negative (not represented). H&E: Hematoxylin and Eosin stain; CK20: Cytokeratin 20; TTF-1: Thyroid Transcription Factor 1; CDX2: Homeobox Protein CDX-2.

A biopsy of the lesion (Figure 2) revealed a neoplasm with an infiltrative growth pattern extending through the full thickness of the dermis. The tumor consisted of cords of atypical cells with marked nuclear pleomorphism and vacuolated cytoplasm, suggesting areas of ductal differentiation. Immunohistochemistry was strongly and diffusely positive for cytokeratin 7 but negative for CK20, TTF-1, and CDX2. This histological and immunohistochemical profile was consistent with adenocarcinoma, which could be primary cutaneous or a skin metastasis of an adenocarcinoma of another organ. Staging investigations, including cervical and breast ultrasound and whole-body positron emission tomography-computed tomography (PET-CT), were performed, all of which showed no suspicious lesions elsewhere in the body. Based on all these findings, a diagnosis of primary cutaneous AANOS was made.

As the disease was confined to the skin with no evidence of metastases, a radical excision was performed down to the level of the periosteum. Histological analysis showed clear peripheral margins; however, the deep margin corresponding to the periosteum showed focal tumor invasion. Given this finding, the patient underwent radiotherapy to the tumor bed (66 Gy in 33 fractions over six weeks).

The patient was recommended a regular follow-up with clinical assessments every three months and PET-CT scans every six months to monitor for recurrence. During this period, he was diagnosed with HIV and started on antiretroviral therapy with emtricitabine, tenofovir, and raltegravir. However, due to prolonged periods working abroad, he was unable to maintain the recommended follow-up schedule and missed several appointments. Consequently, he was lost to follow-up for an extended period. He re-presented three years after the initial treatment with a significant metastatic disease burden. A new suspicious mass was identified in the right mastoid region (adherent to the primary site). A biopsy confirmed a relapse of the adenocarcinoma. Additionally, the patient exhibited significant symptoms, including dyspnea requiring 2 L/minute oxygen in ambulatory settings and a 29% weight loss (from 75 kg to 53 kg). His performance status declined from ECOG-PS 0 to 2, reflecting reduced autonomy.

Re-staging with a PET-CT scan revealed multiple pulmonary lesions. A biopsy of one pulmonary lesion confirmed metastatic adenocarcinoma. Additional findings included mediastinal adenopathy, a voluminous pleural effusion, and multiple blastic bone metastases involving the rib cage, vertebrae, and hip bones.

The case was discussed in our multidisciplinary team, including specialists from dermatology, oncology, and radiotherapy. Given the high tumor burden and severe symptoms with impact on daily life, systemic chemotherapy with paclitaxel and carboplatin was initiated (carboplatin (area under the curve, AUC 2) + paclitaxel 50 mg/m^2^ on days one, eight, and 15 of a 28-day cycle). This treatment resulted in a rapid improvement in symptoms and quality of life, with resolution of dyspnea and oxygen dependency and weight recovery to 74 kg.

**Figure 3:**
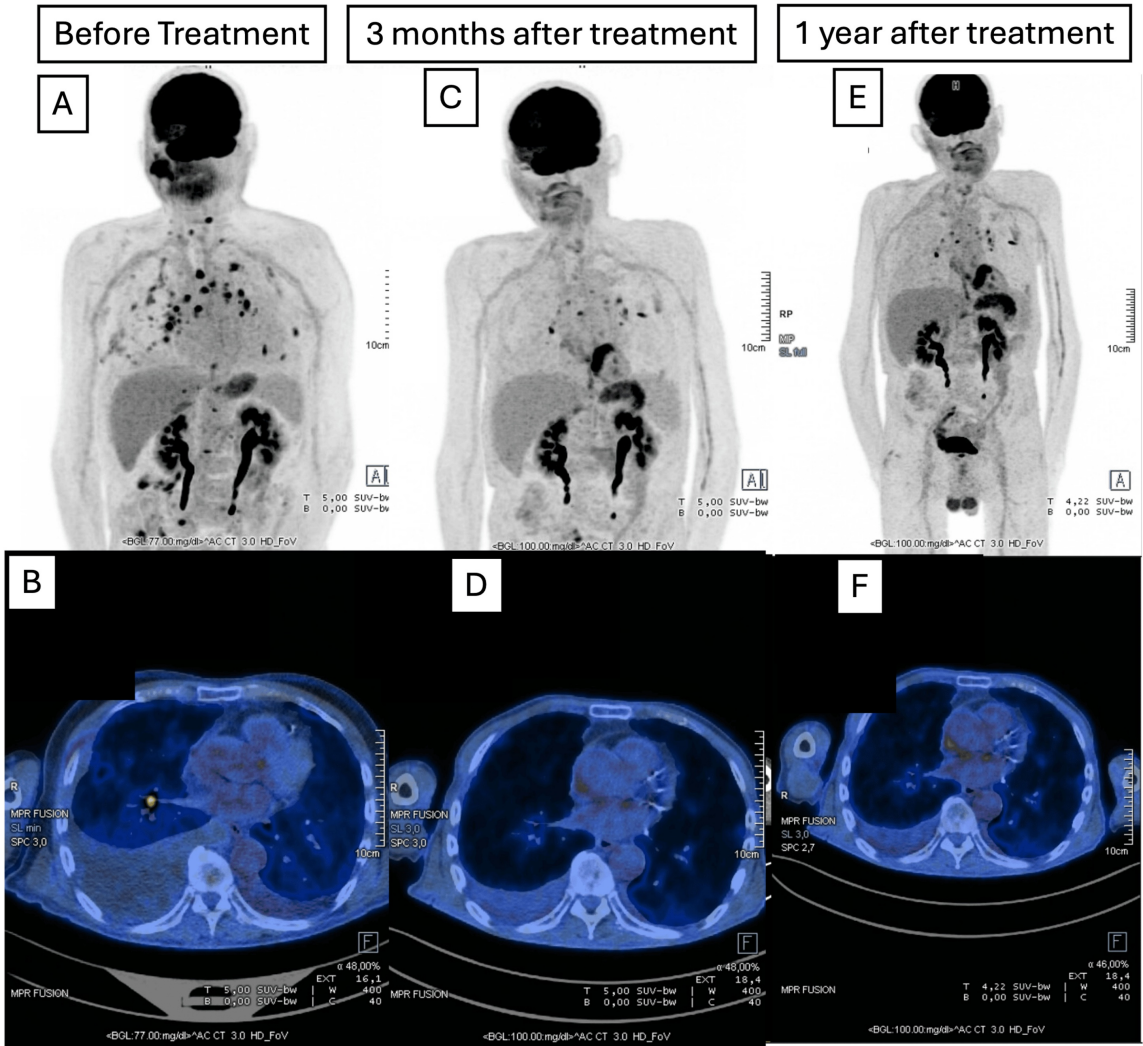
PET-CT scan comparative results: before treatment (A,B), three months (C,D), and one year (E,F) after the initiation of treatment. PET-CT: Positron Emission Tomography-Computed Tomography; AUC: Area Under the Curve.

A PET-CT scan performed after three months (Figure 3) showed a partial response, with complete resolution of the cutaneous mass of the right mastoid region, a reduction in the number and metabolic activity of pulmonary metastases, and complete resolution of bone lesions. A subsequent PET-CT scan after one year confirmed sustained disease control, with findings comparable to those of the three-month evaluation and no evidence of progression.

The patient has now completed one year of treatment without major side effects or dose reductions and will continue therapy until intolerable toxicity or disease progression occurs.

## Discussion

This case shows that carboplatin and paclitaxel can be an effective treatment for metastatic AANOS. Given the rarity of these tumors, no treatment guidelines are available, and evidence is based on single case reports and a few case series [1,5].

Based on the epithelial origin of this neoplasm, conventional chemotherapy may be considered [1,6]. Paclitaxel is a taxane that stabilizes polymerized microtubules of the mitotic spindle to stop mitosis. Carboplatin is a platinum salt that creates cross-links between DNA strands, preventing DNA replication. Given their broad-spectrum efficacy, carboplatin and paclitaxel are used in the treatment of various solid tumors, including cancers of unknown primary.

Alternatives such as targeted therapy [7] and immunotherapy [1] have been used in other settings. Targeted therapy against estrogen receptors has been reported in adnexal tumors but requires testing for estrogen receptor expression and may be less effective in high tumor burden cases and/or highly symptomatic (as in the case described). Immunotherapy, such as immune checkpoint inhibitors, is effective in some metastatic cancers, such as melanoma, but was not optimal here due to the need for a rapid and robust cytotoxic response. Moreover, for this type of tumor, the evidence for immunotherapy is still very low, with only a few cases reported in the literature [1].

The decision to use carboplatin-paclitaxel was guided by the high tumor burden and symptoms in this patient and its ability to quickly control the aggressive disease. Furthermore, this combination allows for the administration of lower doses at a higher frequency, thus balancing efficacy with reduced toxicity.

Relative to radiotherapy, there are no established guidelines for this type of tumor, and some authors suggest that it can be treated similarly to high-risk cutaneous squamous cell carcinoma (SCC) [1]. In such cases, the recommended dose of adjuvant radiotherapy can reach a biologically effective dose (BED) of up to 79.2 (equivalent to 66 Gy with 2 Gy per fraction). In this case, the higher dose was chosen due to the presence of positive margins.

Moving forward, continued research and studies are warranted to further investigate the long-term effectiveness and potential side effects of carboplatin and paclitaxel in the treatment of metastatic AANOS.

## Conclusions

This case represents one of the few documented instances of a significant and sustained response to carboplatin and paclitaxel in the treatment of metastatic AANOS, a rare and aggressive cutaneous tumor. The observed therapeutic outcomes underscore the potential of this combination chemotherapy regimen to achieve effective disease control and substantially improve the quality of life in patients with advanced AANOS. In the absence of established treatment guidelines, this case contributes valuable clinical evidence supporting the use of carboplatin and paclitaxel as a viable systemic therapy option. It also highlights the importance of considering such combinations in managing rare and challenging malignancies.

A key lesson from this case is the critical need for close and consistent follow-up in patients treated with curative intent. Given the high risk of recurrence in adnexal carcinomas, regular clinical evaluations and interval imaging, ideally every three to six months, are essential to detect disease relapse or metastasis at an early stage. This emphasizes the role of long-term monitoring in improving patient outcomes in rare tumors. Further research is needed to validate the efficacy of systemic chemotherapy, including carboplatin and paclitaxel, in similar cases. Expanding the understanding of treatment responses and exploring alternative strategies will be critical to optimizing the management of rare adnexal carcinomas.
